# Advances in the curative management of oesophageal cancer

**DOI:** 10.1038/s41416-021-01485-9

**Published:** 2021-10-21

**Authors:** Jarlath C. Bolger, Claire L. Donohoe, Maeve Lowery, John V. Reynolds

**Affiliations:** Trinity St. James’s Cancer Institute, Dublin 8, Ireland

**Keywords:** Oesophageal cancer, Oesophageal cancer

## Abstract

The incidence of oesophageal cancer, in particular adenocarcinoma, has markedly increased over the last four decades with adenocarcinoma becoming the dominant subtype in the West, and mortality rates are high. Nevertheless, overall survival of patients with oesophageal cancer has doubled in the past 20 years, with earlier diagnosis and improved treatments benefiting those patients who can be treated with curative intent. Advances in endotherapy, surgical approaches, and multimodal and other combination therapies have been reported. New vistas have emerged in targeted therapies and immunotherapy, informed by new knowledge in genomics and molecular biology, which present opportunities for personalised cancer therapy and novel clinical trials. This review focuses exclusively on the curative intent treatment pathway, and highlights emerging advances.

## Introduction

Oesophageal cancer, comprising mainly oesophageal adenocarcinoma (OAC) and squamous cell carcinoma (SCC), is the seventh most common cancer worldwide, and is responsible for ~450,000 deaths per year [1]. While historically viewed as a cancer with a dismal prognosis, encouraging trends have emerged across several domains. First, reports of the International Cancer Benchmarking Partnership (ICBP-SURVMARK-2 project), which compare the 1995–1999 to 2011–2014 time periods, highlight an approximate doubling of 5-year survival for both OAC and SCC across seven high-income countries, with the greatest impact in patients under 75 years of age [2, 3]. Second, in an era in which combination therapies rather than surgery alone are standard in the curative approach to patients presenting with locally advanced disease, a modern benchmark for 5-year survival approaches 50%, which is also an approximate doubling over a 20 year time period [4–6]. Third, an increase in early detection rates of mucosal and submucosal lesions through a combination of greater cancer awareness, surveillance of Barrett’s oesophagus (a precursor to oesophageal cancer), management of gastro-oesophageal reflux disease (GORD; a risk factor for Barrett’s oesophagus and, hence, for oesophageal cancer) and advances in staging has permitted the relatively low-risk treatment of selected patients with endotherapy approaches (endoscopic eradication therapy, EET), such as endoscopic mucosal resection (EMR), endoscopic submucosal dissection (ESD) and radiofrequency ablation (RFA) (Box 1) [7–9].

Furthermore, advances in genomics and molecular research, and the advent of immunotherapeutic and targeted approaches, have improved our understanding of oesophageal carcinogenesis and tumour biology and enabled the development of novel approaches that could improve outcomes [10–13]. Finally, advances in surgery, including standardisation of the extent of resection and lymphadenectomy, and improvements in perioperative care, and in the array of approaches, including minimally invasive and robotic-assisted techniques, have presented opportunities to improve both oncological and operative outcomes [14–16].

Despite these clinical and scientific advances, however, several concerns exist in everyday multidisciplinary team discussions and decision-making regarding patients who can be treated with curative intent. These concerns include the criteria for endotherapy, the choice of neoadjuvant therapy, differential considerations for SCC and OAC, the consideration of a non-operative surveillance approach in patients with an apparent complete clinical response to neoadjuvant therapy, the approach to surgery, and the role of immunotherapy and targeted therapy. This review focuses exclusively on these themes in the curative intent treatment pathway, and discusses existing standards, emerging advances and key controversies. The authors acknowledge significant developments in staging, assessment of treatment response, perioperative care, targeted radiation and proton therapy, surgery for oligometastatic disease and diagnostics, and refer the readers to relevant reviews on these topics [17, 18] however, owing to word limitations and our clinical and academic focus, we have structured and prioritised this review into five major sections.

Box 1: Endotherapies currently in widespread use in oesophageal cancer

**Table:** 

Endotherapy	Use	Benefits	Drawbacks
Radiofrequency ablation	Medium frequency alternating current is used to ablate Barrett’s mucosa with low- or high-grade dysplasia	Potential for eradication of Barrett’s Prevent progression to invasive malignancy Prevent morbidity and mortality previously associated with oesophagectomy for dysplasia	Requirement for multiple sessions Potential for stricture formation Potential to miss submucosal lesions
Endoscopic mucosal resection	Areas of mucosa are sucked into endoscope cap, snared, resected and sent for histological analysis	May allow complete resection of early cancers (T1a m1-3 and sm1) May act as a staging procedure May allow resection of nodules and then progression to RFA	Complications include bleeding and perforation Potential for stricture May need multiple EMRs Needs close surveillance Does not estimate nodal burden
Endoscopic submucosal dissection	Lesions are raised with submucosal injection then resected en-bloc and sent for histological analysis	Allows en-bloc resection of lesions Allows more accurate assessment of margins and potential invasion	Technically challenging Higher risk of bleeding and perforation compared with EMR
Chromoendoscopy	Use of staining agents such as acetic acid or Lugol’s iodine to show abnormal areas of mucosa	Allows detection and monitoring of early mucosal lesions	Inter-observer variability

## Endotherapy for mucosal and submucosal cancer

**Fig. 1:**
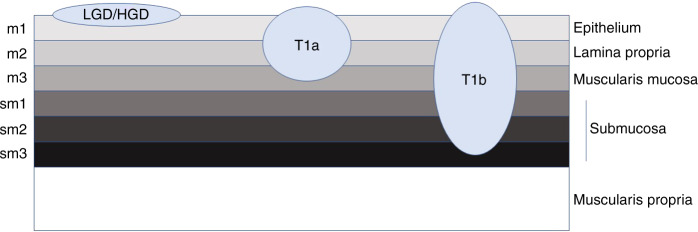
Overview of the staging of early oesophageal cancers based on their depth of invasion. Low-grade and high-grade dysplasia are confined to the epithelium. T1a tumours are separated based on their depth of invasion (m1-3). T1b tumours invade into the submucosal layer and are further subdivided based on depth of invasion (sm1-3).

Figure 1 gives an overview of staging of early tumours as regards their depth of invasion.

The incidence of OAC has increased in parallel with the rising incidences of GORD and Barrett’s oesophagus in Western populations. As access to endoscopy improves and hence a lower threshold for endoscopy and technological improvements have resulted in an increase in the detection of dysplastic lesions and early cancers [19, 20]. Furthermore, in the East, the high incidence of SCC of the oral cavity, pharynx and oesophagus, together with strict surveillance policies, is conducive to the diagnosis of early lesions. The assessment and management of apparent mucosal and submucosal disease is enhanced by chromoendoscopy using acetic acid or Lugol’s iodine, virtual chromoendoscopy and targeted biopsy protocols [20].

Whereas an oesophagectomy was once the standard approach for patients with high-grade dysplasia (HGD) or mucosal invasion (T1a), the rarity of lymph node metastasis (<2%) in this context supports a more targeted local approach. Accordingly, in the latest American Gastroenterological Association guidelines, EET, in the form of EMR and ESD, is preferred to continuing surveillance or oesophagectomy [8, 9]. The current treatment algorithm reserves oesophagectomy for patients with T1b classification, denoting submucosal invasion, in which lymph node metastases occur in approximately 20% of cases, and for multifocal carcinoma or for lesions that are not amenable to endoscopic resection [9, 19]. Thus, the ability to easily differentiate mucosal (T1a) from submucosal (T1b) invasion would be of value. However, both endoscopic ultrasonography and CT-PET are limited by low sensitivity [17, 21].

## Endoscopic features

**Fig. 2:**
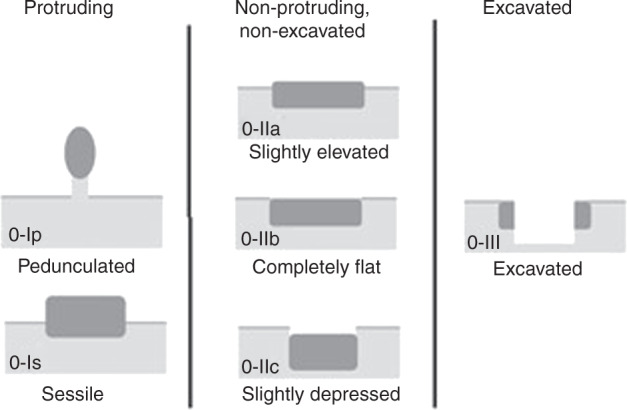
The Paris endoscopic classification system describes the lesions as protruding, excavated or flat (non-protruding, non-excavated). This system has not been validated as a prognostic tool in Barrett’s oesophagus but studies suggest that sessile and depressed lesions are more likely to contain invasive cancer, with IIa and IIb lesions at higher risk of associated invasive malignancy.

Endoscopic features help to guide the selection of patients for EMR and ESD. The Paris classification (Fig. 2) was developed from previous Japanese classifications, and describes lesions as protruding, excavated or flat (neither protruding nor excavated). It includes type 0–I, which denotes elevated or polypoid forms (pedunculated (Ip) and sessile or broad-based (Is)); type 0–II, flat or superficial (IIa flat and elevated, IIb completely flat, and IIc superficially depressed); and type 0–III, indicating excavated or ulcerated [22, 23]. Type III lesions and, to some degree, type IIc, might be associated with more aggressive tumour growth, and ulcerated lesions usually reflect deeper level disease that is less suitable for endoscopic therapy for technical reasons as well as being a proxy of understaging.

## EET for OAC

When endoscopic resection is considered technically feasible, EMR has become the standard of care for OAC (T1a-m1–3-sm1, early tumours confined to the mucosa or most superficial third of the submucosa) in the West, with ESD preferred in Japan—at minimum to establish a T classification, but in many cases as definitive therapy [8, 9]. ESD is increasingly utilised for larger suspicious lesions where *en bloc* EMR is not possible [23, 24]. Modern criteria for curative resection include negative lateral and deep margins (R0), absent lymphatic or vascular invasion (LVI), G1 or G2 grade, well or moderately well differentiated, and absent penetration beyond the first (SM1) layer of the submucosa, approximating to <500 µm depth. Endoscopic resection should be followed approximately 8 weeks later by biopsies of the tumour bed as well as mapping biopsies (mucosal biopsy samples taken sequentially from proximal to distal) of the entire at-risk segment, from 10 mm above the squamo-columnar junction to 5–10 mm distal to the Z line of the oesophagus. RFA is commonly used in combination with endoscopic surgery, although photodynamic therapy, argon beam coagulation or cryoablation might also ablate surrounding at-risk epithelium [7]. With such interventions, the recurrence rate is reported to be between 4.5% and 14.5%, with a median time to recurrence of approximately 2 years, which supports regular endoscopic surveillance typically at 3, 6 and 12 months.

## EET for SCC

Endoscopic therapy is less evolved for SCC. The Japanese Esophageal Society recommends the use of vessel irregularities, such as the loss of loop formation and the presence of dilated and tortuous vessels, as a guide to predict the depth of invasion [25]. When combined with conventional endoscopy, magnifying blue laser imaging has been shown to be superior for determining the depth of invasion; moreover, it has low inter-observer variability [26]. Endoscopic surgery for SCC is best studied in Japan—consequently, most of the literature relates to ESD rather than EMR. Early lesions can be resected, assuming that they are less than 5 cm in axial dimensions and not completely circumferential, as can all lesions with B1 vessel pattern (predicted infiltration T1a superficial to muscularis mucosa), and a B2 vessel pattern, suggesting extension to muscularis mucosa or the superficial submucosa (SM1) [25, 26]. R0, G1/G2 grade, and absent LVI are good prognostic indicators, and superficial infiltration of the submucosa (≤200 µm) is acceptable in selected cases [27, 28]. An involved margin and adverse pathology such as LVI indicate the need for additional treatment—either an oesophagectomy or adjuvant chemoradiotherapy. A series of 176 patients who initially had ESD, of whom 87 had pT1a with LVI and the remainder had pT1b tumours, and who received adjuvant chemoradiotherapy, reported an excellent 3-year survival of 90%, thus presenting this approach as a valid alternative to oesophagectomy [29].

## Optimum approach to locally advanced OAC and SCC

The seminal randomised clinical trial (RCT) examining the use of neoadjuvant therapy in oesophageal cancer, a modern benchmark for subsequent trials in OAC and SCC, is the CROSS Trial, a multicentre Dutch study of 366 patients, 75% of whom had OAC, which recruited from 2004 to 2008, and published initially in 2012 [5]. This RCT conclusively established that neoadjuvant chemoradiotherapy (paclitaxel, carboplatin and 41.4 Gy/23 fractions) prior to resection was superior to surgery alone in patients with locally advanced cancer. Median survival was 45 months for OAC and 81.6 months for SCC, compared with 24 months in the surgery-only group. The overall 5-year survival was 47% with multimodal therapy (neoadjuvant therapy followed by definitive surgical resection), and no evidence of added operative mortality or major morbidity was evident. In spite of the outstanding outcomes, key concerns persist—in particular, whether this approach is superior to perioperative chemotherapy without radiation, and whether definitive chemoradiotherapy is a valid alternative to perioperative chemotherapy. Due to differences in tumour biology, which are particularly relevant in terms of the response to radiation therapy, each pathological type of oesophageal cancer will be discussed separately.

## OAC

In some parts of the World—particularly the UK, France and Germany—chemotherapy prior to and after surgery was the dominant approach prior to the CROSS trial, and subsequently has been slow to change.

### Challenging the CROSS trial

This reluctance to change is, to a large extent, due to level I evidence (in the UK and France) from RCTs of gastric adenocarcinoma and OAC predating CROSS which clearly established the superiority of perioperative chemotherapy compared with surgery alone [30, 31]. In addition, some reports of poor operative outcomes following preoperative radiation therapy at that time highlighted safety concerns about potential added operative risk [32]. The MAGIC RCT, designed for gastric and gastro-oesophageal junction tumours, and latterly including oesophageal tumours, arguably had the greatest impact in changing treatment approaches. Three cycles of epirubicin, cisplatin and fluorouracil before and after surgery resulted in a 5-year survival of 36% in the treated arm compared with 23% for surgery alone [30]. A similar finding was observed in the contemporaneous French Accord trial, in which 75% of patients had junctional or oesophageal adenocarcinoma, with a 5-year survival of 38% in patients receiving perioperative therapy compared with 24% in those undergoing surgery alone [31]. In the UK Medical Research Council OE05 RCT, published in 2017, 897 patients from 78 UK centres were randomised to receive either two cycles of cisplatin and fluorouracil or four cycles of epirubicin, cisplatin and capecitabine preoperatively, with surgery standardised to a two-stage radical en bloc resection. Median survival was 23.4 (95% CI, 20.6–26.3) and 26.1 (22.5–29.7) months, respectively, thus providing no added rationale for triplet therapy [33].

In this overall context, results from the German FLOT-4 RCT, published in 2019, look set to be a game changer globally and have already challenged existing patterns of care [4]. In this Phase II/III RCT, 716 patients with gastric, lower oesophageal or gastro-oesophageal junction adenocarcinoma received either a modified MAGIC protocol or the FLOT regimen of fluorouracil plus leucovorin, oxaliplatin and docetaxel administered as four pre-operative and four post-operative cycles. The median survival of 50 months versus 35 months in favour of FLOT was particularly impressive, and a pathological complete response (pCR) rate of 16% compared with 6% for the control (modified MAGIC protocol) group approached published pCR outcomes using neoadjuvant chemoradiation [6]. The sole, but major, caveat was a high incidence of grade 3 or 4 neutropenia, at 51%, and severe infections, at 18%, and just 46% of patients completed all cycles of chemotherapy. A case series (*n* = 131) in patients with OAC also reported impressive outcomes in response to FLOT, with a major pathological response rate (MPR) of 31%, a 5-year survival of 51%, and a 35% incidence of recurrence (with just 16% being locoregional recurrence) [34].

### Multimodal therapy versus chemotherapy

Accordingly, whether neoadjuvant chemoradiotherapy, best represented by the CROSS regimen, is superior to optimum perioperative chemotherapy, with FLOT increasingly being seen as the standard, is of major current interest. Previous studies, including the Phase III POET RCT, which closed early, a Phase II RCT (NeoRES-1), and a small Australasian RCT, failed to show any survival advantage using multimodal protocols compared with perioperative chemotherapy [35–37] (Table 1). In NeoRES-1, the 3-year survival was 47% versus 49%, *P* = 0.77, in multimodal versus chemotherapy alone, respectively, despite higher pCR rates (28% versus 9%) and lower numbers of pathologically (ypN0) involved nodes [36] (38% vs 65%, *P* = 0.001). An important further caveat was the occurrence of increased perioperative mortality rates, at 6% vs 3% for NeoRES and 10.2% vs 3.8% for POET for multimodal versus chemotherapy alone,^,^ respectively [36, 37].

**Table 1:** Reported randomised controlled trials (RCTs) in patients with OAC, highlighting equivalent overall survival following chemotherapy only or multimodal regimens, despite higher pathological complete response (pCR) rates and negative resection (R0) margins with multimodal therapy.

Trial	Comparison	Overall survival arm A	Overall survival arm B	R0	pCR
POET [37] (*n* = 119)	Arm A: CF/w x 14 versus Arm B: Same + 30 Gy+ CEt	24% 5 years	40% 5 years, *P* = 0.0055	79% versus 88% 41/52 versus 43/49	2% versus 12% (1/59 versus 7/60), *P* = 0.03
AUSTRALIAN [35] (*n* = 75)	Arm A: CF/q21d x2 versus Arm B: Same + 35 Gy	36% 5 years	45% 5 years	88% versus 100% 29/33 versus 33/33	0 versus 13% (0/36 versus 5/39), *P* = 0.05
NEORES I [36] (*n* = 181) 72% Adeno	Arm A: CF/q21d x 3 versus Arm B: Same + 40 Gy	49% 3 years	47% 3 years	74% versus 87% 58/78 versus 68/78, *P* = 0.042	8% versus 24% (7/91 versus 22/90), *P* = 0.002

*C* cisplatin, *F* fluorouracil, *Et* etoposide, *E* epirubicin.

**Fig. 3:**
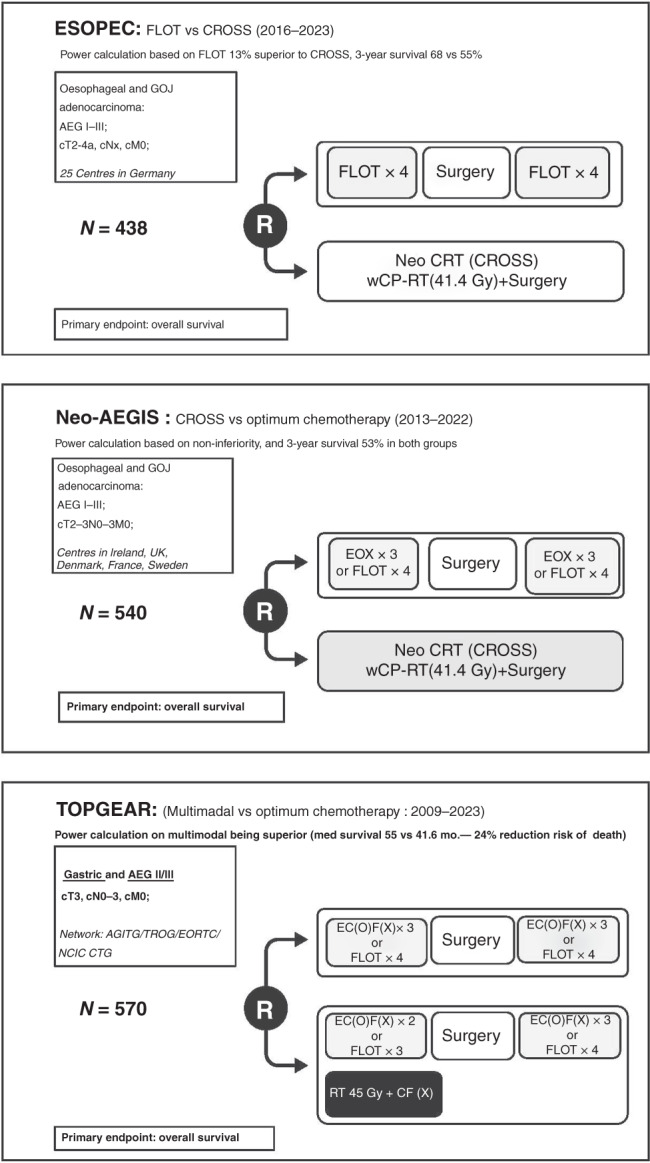
Key ongoing trials of perioperative and neoadjuvant therapy for oesophageal adenocarcinoma. FLOT fluorouracil, leucovorin, oxaliplatin and docetaxel, CROSS paclitaxel, carboplatin and 41.4 Gy/23 fractions, EOX epirubicin, oxaliplatin, capecitabine, EC(O)F(X) epirubicin, cisplatin (or oxaliplatin), fluorouracil (or capecitabine).

Several major, well-designed, Phase III RCTs are currently active and will address whether a multimodal approach is superior to chemotherapy alone (Fig. 3). ESOPEC is a multicentre German RCT (*n* = 428) directly comparing FLOT with CROSS for OAC, with a power calculation based on FLOT being 13% superior to CROSS [38]. Neo-AEGIS (*n* = 540) is a European RCT that initially used a modified MAGIC regimen (EOX) as the control arm versus CROSS, but since 2018 has permitted the use of FLOT [39]. An initial power calculation for 10% superiority to CROSSwas adjusted for non-inferiority after the initial futility analysis. TOPGEAR (*n* = 570) compares perioperative chemotherapy (modified MAGIC but FLOT permitted since 2017) with neoadjuvant chemoradiotherapy (45 Gy and cisplatin/fluorouracil (capecitabine, a pro-drug of 5-FU) in gastric and oesophageal adenocarcinoma, and is powered on superiority of the multimodal arm [40]. These trials include over 1000 randomised patients and will provide considerable novel data, and should determine whether there is a superior arm and new gold standard, or whether the approaches are equivalent. Important secondary endpoints include recurrence patterns, postoperative morbidity and mortality rates, and treatment-related toxicity. Irrespective of the overall conclusions, the limitations of these RCTs will be specific clinical scenarios that might be excluded, such as cT4a, as well as the optimum management of cN3, ypN1–3 post neoadjuvant chemoradiotherapy, and signet ring cell carcinoma/diffuse pathological subtypes as distinct from the more common intestinal pathological subtype. Another RCT on this theme, RACE, has commenced in Germany; it is designed as a prospective, randomised, stratified Phase III clinical trial comparing standard FLOT with FLOT plus pre-operative radiation therapy using a 45 Gy dose [41]. This RCT aims to recruit 340 patients, with progression-free survival as the primary endpoint.

### Optimum radiation therapy plus chemotherapy

Another multimodal strategy is to explore optimum combinations of radiation therapy with chemotherapy in the neoadjuvant setting. NEOSCOPE randomised 85 patients to receive either neoadjuvant oxaliplatin-capecitabine or carboplatin-paclitaxel with 45 Gy concurrent radiation therapy, and reported a higher pCR rate in the carboplatin-paclitaxel group [42]. The PROTECT-1402 compares FOLFOX plus radiation with the CROSS regimen [43]. The aim is to recruit 106 patients, with the primary outcome being R0 resection rates. In the Alliance Trial, patients (*n* = 257) were randomised to a modified FOLFOX or carboplatin–paclitaxel combined with radiotherapy (50.4 Gy/28 fractions) [44]. Interestingly, if a patient was deemed to be a non-responder by a second CT-PET [ <35% decrease in standardised uptake value (SUVmax)], he/she crossed over to the other chemotherapy regimen. The 2-year overall survival was 61.8% (95% CI 55.7–68.5%), with a median of 40.2 months for responders and 27.4 months for non-responders, and the best outcomes in responders were with the FOLFOX combination therapy. This RCT also highlighted the positive predictive impact of a CT-PET-determined metabolic response on prognosis and how it might be adapted to a trial design, as reported originally in the MUNICON trials [45–47].

## SCC

Although most trials on oesophageal cancer have included OAC and SCC patients together, the pCR rates after neoadjuvant chemoradiotherapy of 49% versus 23% for SCC versus adenocarcinoma, respectively, in the CROSS trial illustrate the higher sensitivity of SCC to radiation regimens [5, 6]. Although the number of treated SCC patients was small (*n* = 41), the impressive response rate and survival has established a modern benchmark. In a national, high volume centre, multimodal therapy was associated with a 5-year disease-specific survival of 62% in a series of 75 patients with SCC [48]. An international RCT (NEOCRTE5010) of 451 patients with SCC showed a median survival of 100 months versus 66.5 months in favour of multimodal therapy, with a pCR rate of 43.2% (*P* = 0.025) [49].

### Differing treatment approaches

However, despite the apparent success of multimodal therapy for SCC patients, there are contrary views on this topic, and practice varies widely. The East has seen a gradual shift in the SCC treatment algorithm from adjuvant therapy to neoadjuvant approaches, with a preference largely for chemotherapy alone, and with an increasing use of docetaxel, a key component of the FLOT regimen. Reported response rates are over 60% [50, 51]. Currently, an RCT (JCOG1109, NExT study) conducted by the Japan Esophageal Oncology Group has completed recruitment with a view to comparing three neoadjuvant therapy regimens: cisplatin and 5-fluorouracil versus cisplatin, 5-fluorouracil and docetaxel versus cisplatin, 5-fluorouracil and radiation therapy (41.4 Gy) [52]. In the West, conversely, the debate largely relates to multimodal therapy versus definitive chemoradiotherapy, and both are accepted as equivalent within international guidelines and in the Cochrane database of systematic reviews [53]. This judgement of equipoise is largely based on two RCTs. A German trial (*n* = 189) published in 2005 compared chemoradiotherapy (cisplatin, etoposide-40 Gy) followed by surgery with continued chemoradiotherapy increasing to 65 Gy in patients with cT3 and cT4 tumours, with all patients having had initial induction chemotherapy with fluorouracil, leucovorin, etoposide and cisplatin [54]. Those undergoing surgery demonstrated an improved 2-year progression-free survival (64% versus 40%, *P* = 0.003) compared with those receiving continued CRT, but showed no improvement in overall survival and a high in-hospital mortality rate (11.3%). A French RCT (FFCD 9102) similarly showed no difference in survival in patients treated with induction therapy (cisplatin and 5-fluorouracil-RT) and randomised, if responding, to either continued chemoradiotherapy or surgery; however, locoregional relapse was higher in the chemoradiotherapy group [55]. Conversely, both RCTs reported a significant increase in treatment-related mortality in the multimodal cohorts [55, 56]. Accordingly, no level I data have established a standard of care for locally advanced SCC globally. Nevertheless, the impressive outcomes from CROSS and other series of multimodal therapy and, more recently, data analysis of the (US) National Cancer Database of 19,532, which reported a doubling of survival for multimodal compared with definitive chemoradiotherapy in non-randomised comparisons, provides support for an approach that includes surgery as a standard of modern practice, particularly in the modern era in which the risk of operative mortality is low [56]. In Japan, although preoperative chemotherapy is more standard than multimodal therapy, a JCOG trial (0909) of 50 Gy RT with cisplatin and 5-fluorouracil in SCC Stage II/III patients resulted in excellent outcomes, and this regimen is currently used as standard therapy in patients who do not wish to have surgery or are relatively unfit [57].

### Influence of radiation dose

What of higher doses of radiation in the definitive chemoradiotherapy setting? An established protocol involves 50.4 Gy, particularly in North America, and no clear evidence exists of the benefit of increasing doses [58, 59]. The ARTDECO (61.6 vs 50.4 Gy) and CONCORDE/PRODIGE 26 (76 vs 50 Gy) trials compare higher doses to standard doses of radiation therapy [60, 61]. In 2020, ARTDECO reported no improvement (*P* = 0.08) in local progression-free survival with a higher dose of radiation therapy, and an increase in grade 4/5 toxicity [61]. In the UK, SCOPE 2 (NCT02741856) is examining dose escalation from 50 to 60 Gy in definitive chemoradiotherapy [62].

### Alternative approaches

Because SCC is less common in Western patients, a proposed RCT with surgery as part of the control arm has struggled to recruit sufficient eligible patients [63]. If definitive chemoradiotherapy is preferred based on application of guidelines, centre preference, patient fitness or preference, then some patients who develop recurrence locally might be suitable for so-called salvage oesophagectomy [56]. Encouragingly, in a large population study, there was no significant difference in median survival for those who underwent neoadjuvant chemoradiotherapy plus surgery compared with those with local recurrence undergoing salvage oesophagectomy (36 months versus 35.5 months, *P* = 0.8) [56]. Perioperative outcomes were also similar, with no difference in perioperative mortality. This compares favourably with prior series reporting mortality rates of up to 25% in those undergoing salvage procedures [64]. Finally, proton beam therapy (PBT) is emerging as an interesting alternative to standard radiation therapy regimens, although trial data is currently lacking [65]. PBT offers the potential for reducing off-target side effects while maintaining the dose distribution to the primary tumour. Early work suggests is may be efficacious in OAC and SCC.

## Surgery as needed (‘watch and wait’) for complete clinical responders after neoadjuvant therapy

Although salvage surgery is used after definitive chemoradiotherapy for isolated local recurrence, or persistent disease, an emerging concept is whether patients with apparent complete response to neoadjuvant therapy prior to planned surgery can be kept under strict surveillance and only operated on if proven locoregional disease is evident in follow-up. The advantages are clear, and include the avoidance of a major operation with an up to 5% risk of mortality (as well as attendant major morbidity), in addition to organ preservation. This concept might have gained extra importance during the SARS-CoV-2 pandemic, which has led to alterations in standard care pathways and a pragmatic consideration of such an approach [66].

## Assessing the ‘watch and wait’ approach

The ‘watch and wait’ rationale appears more compelling for patients with SCC, almost 50% of whom had a pCR in the CROSS Trial, compared with 25% of patients with OAC [5]. However, evidence is needed to prove that a complete clinical response equates to a pCR and, to this end, the PreSANO (Surgery as Needed for Oesophageal Cancer) study was designed [67]. PreSANO was a single arm, multicentre study from six centres in the Netherlands, and included patients with OAC and SCC. In the design, patients with an apparent complete clinical response based on endoscopic assessment, bite-on-bite (deep) biopsies and endoscopic ultrasonography at 6 weeks had a further clinical response evaluation at 12 weeks, which included CT-PET, endoscopic ultrasonography plus fine needle aspiration of suspicious nodes, and additional biopsies. The study reported good sensitivity (90%) for detecting residual disease (>10% vital residual tumour cells) but poor sensitivity for detecting the complete absence of residual disease, with residual disease often microscopic and confined to the submucosa [67]. A presentation Van Der Wilk B, et al. [68] at the 2020 International Society of Diseases of the Esophagus (ISDE) Meeting involving 256 patients from seven studies undergoing a watch and wait approach reported a pooled 5-year survival of 58%, with locoregional recurrence of 33% at 2 years. The limited sensitivity shown in preSANO and other series for individual elements of clinical response evaluation—in particular CT-PET and endoscopic ultrasonography—presents a notable caveat with respect to pursuing this ‘watch and wait’ approach outside of RCTs, particularly for OAC [69–71]. The preSANO group has activated the SANO trial, which will have as a primary endpoint non-inferiority of a strict surveillance programme versus surgery, if the 12-week clinical response evaluation shows no residual disease [72]. The French Esostrate-Prodige 32 study is similar in design to SANO, but the randomisation method differs [73]. For SCC alone, in four Asian Centres, a preliminary study preSINO (surgery if needed for oesophageal cancer) is also underway based on preSANO principles; this might progress to an RCT [74].

## Multimodal therapy versus definitive chemotherapy and potential salvage surgery

Although patients might accept some risk in avoiding surgery, potential downsides exist, as intensive surveillance brings with it the necessity for frequent hospital attendance for procedures, with inherent anxiety and impact on quality of life, in addition to an anticipated requirement for ‘salvage’ surgery in approximately one in three patients [75]. The NEoadjuvant chemoradiotherapy for Esophageal squamous cell carcinoma versus Definitive chemoradiotherapy with salvage Surgery as needed (NEEDS trial-EudraCR 2020-000149-15) is an important new international RCT, led by the Karolinska Institute, that has recently commenced recruitment. The trial compares CROSS regimen multimodal therapy with definitive chemoradiotherapy and salvage surgery as needed, and is powered for non-inferiority, with a target to recruit 1200 patients and an estimated study completion date of 2031 [76].

## Immunotherapy and targeted therapies in the neoadjuvant and adjuvant context

Advances in cancer genomics, molecular biology and immunology are bringing about a modern revolution in cancer therapy [10–13]. In the context of oesophageal cancer, this revolution is mainly evident in metastatic or advanced incurable disease, where several novel approaches are approved in first, second and third-line protocols.

## Targeted therapies

Key trials of targeted therapies include TOGA, in which trastuzumab, a monoclonal antibody that targets HER2, in combination with chemotherapy improved outcomes compared with chemotherapy alone in patients with HER2^+^ tumours; and REGARD and RAINBOW, in which the vascular endothelial growth factor (VEGF) receptor inhibitor ramucirumab was beneficial as a single agent or combined with paclitaxel, respectively [77–79]. PETRARCA reported improved disease-free survival and overall survival with the addition of trastuzumab and another HER2-targeting monoclonal antibody, pertuzumab, to the FLOT regimen in patients with HER2^+^, resectable oesophagogastric cancer in a Phase II RCT, with a remarkable pCR of 35% for the combination therapy [80].

## Immunotherapies

Immune therapies that target ‘immune checkpoints’ such as programmed cell death protein 1 (PD-1) and its ligand PD-L1, or cytotoxic lymphocyte antigen 4 (CTLA-4) are currently of great interest [81]. A 10–15% response rate to immune therapies has been reported in patients with recurrent or metastatic oesophageal cancer [82]. Advances in the use of immune checkpoint inhibitors for oesophageal cancer have come from combination studies in the first-line setting. In KEYNOTE-590, a combination of the PD-1 inhibitor pembrolizumab with chemotherapy demonstrated a survival benefit compared with chemotherapy alone in patients with OAC or SCC, particularly in tumours with a combined positive score (the CPS, based on the number of PD-L1 positive cells in relation to the total number of tumour cells) ≥10 [83, 84]. In CHECKMATE-649, in which patients with gastric cancer and OAC were randomised to receive either chemotherapy or chemotherapy plus nivolumab, an overall survival benefit with the addition of the PD-1 inhibitor was seen, particularly in patients with a CPS ≥ 5 [85]. Key monotherapy trials include ATTRACTION-3, an international study in patients with advanced SCC, which reported a survival advantage for nivolumab compared with investigators’ choice chemotherapy in the second-line setting, while KEYNOTE 181 demonstrated a benefit with pembrolizumab over chemotherapy in the second-line setting for patients with SCC and a CPS ≥ 10, but not for patients with OAC [86]. Interestingly, a combination of pembrolizumab and trastuzumab with chemotherapy resulted in excellent progression-free survival in HER2^+^ patients, and is being evaluated in a randomised Phase III trial [87]. The optimal biomarkers of response to immune checkpoint inhibition have yet to be defined; in addition to PD-L1 overexpression, microsatellite instability (MSI), high mutational burden and defects in DNA mismatch repair might also predict a durable response [81].

## Targeted therapies and immunotherapy in the curative setting

Do these developments in treatments for metastatic and incurable disease translate into improved outcomes in curative therapy? In the same way that FLOT is a potential game changer for OAC, adjuvant immunotherapy might hold similar future promise for both OAC and SCC. In the CHECKMATE-577 RCT, 794 patients with Stage II/III oesophageal cancer were treated with neoadjuvant chemoradiation; patients who did not achieve a pCR following resection were randomised 2:1 to receive adjuvant nivolumab or placebo [88]. At an interim analysis, median disease-free survival was doubled (22.4 versus 11 months, HR 0.69 [CI:0.56–0.86) *P* = 0.0003) in patients who received nivolumab. This finding is not only exciting, but scientifically plausible, as radiation therapy induces the expression of PD-L1 and the recruitment of immune cells in the tumour microenvironment [89, 90].

CHECKMATE-577 provides a compelling evidence base that supports immunotherapy in the postoperative adjuvant setting after neoadjuvant chemoradiotherapy, and is a significant advance in adjuvant therapy for oesophageal cancer. Whether this approach is effective in the neoadjuvant context is unknown, and whether adjuvant therapy complements the effect of optimum perioperative chemotherapy regimens such as FLOT also requires evaluation. Studies are active using pembrolizumab, durvalamab (an anti PD-L1 mAb), and tremelimumab (an anti-CTLA-4 mAb) in this context, as are trials combining chemotherapy regimens, including FLOT with immunotherapy or approved targeted therapies [91]. These combination regimens include FLOT-8/DANTE (NCT03421288), which compares the standard perioperative FLOT regimen plus the PD-L1 inhibitor atezolizumab, and FLOT-7/RAMSES (NCT02661971), which uses the FLOT regimen plus ramucirumab [90].

### Neoadjuvant immunotherapy and healing

Another important issue relates to whether immunotherapy can be given safely in the neoadjuvant setting without increasing complications associated with oesophageal surgery—in particular, wound and anastomotic healing—or the postoperative systemic or organ-specific inflammatory response. Relevant data with respect to approaches that impact on inflammation and healing might come from the UK MRC ST03 trial, which randomised 1063 patients with gastric, oesophageal or gastro-oesophageal junction adenocarcinoma to receive epirubicin, cisplatin and capecitabine alone or in combination with the VEGF inhibitor bevacizumab. This trial reported an equivalent 3-year survival of 50.3% (95% CI 45.5–54.9) and 48.1% (95% CI 48.1% (43.2–52.7), respectively, but, of concern, a more than doubling of anastomotic leaks (24% versus 10%) was seen in patients who underwent an oesophago–gastric anastomosis; consequently, recruitment was closed to this cohort based on this analysis [92]. Currently, with limited data published, there is no evidence that immunotherapy in the neoadjuvant context impacts on key operative outcomes; however, this scenario should be carefully studied in prospective evaluation [93, 94].

## Advances in surgery in a minimally invasive era

Open surgery has been the mainstay of surgery for generations, with the main issues in the context of oesophageal cancer being the value of transthoracic oesophagectomy (TTO) compared with a transhiatal approach (TH), which avoids the need for a thoracotomy, and also whether TTO is superior to an extended total gastrectomy and mediastinal anastomosis for adenocarcinoma of the cardia (Siewert type II) [95, 96]. TTO comprises a two-stage (Ivor Lewis) oesophagectomy with an intrathoracic anastomosis or a three-stage oesophagectomy with a cervical anastomosis. One of the advantages of TTO over TH is the extent of lymphadenectomy that is possible within the thorax, and *en bloc* resection along tissue planes on the aorta and tracheobronchial tree. TTO allows more accurate lymph node staging due to the nodal yield, and as dissection from surrounding structures is done under direct vision offers the ability to improve circumferential resection margin rates.

## Minimally invasive approaches to oesophagectomy

Consistent with trends across surgery as a whole, the modern era has seen an increase in minimally invasive and robotic-assisted approaches, with emerging evidence suggesting oncological equivalence compared with open surgery, but perhaps an association with less major morbidity [97]. Although minimally invasive oesophagectomy (MIO) was first reported in the early 1990s, it is in the last decade that it has developed internationally and begun to supplant open approaches [98, 99].

Although logic compels the use of MIO compared to open surgery if it can be safely conducted, RCTs are the gold standard to demonstrate superiority or non-inferiority; however, the evidence base to date is limited, and focuses mainly on the occurrence of postoperative pulmonary complications. The key trials are outlined in Table 2. The Dutch TIME trial compared a completely minimally invasive approach with open surgery in 115 patients [100]. The primary outcome—post-operative microbiologically proven respiratory infection within 14 days—was significantly different, at 9% in the MIO cohort versus 29% in the open group (*P* = 0.05) [14, 100]. The French MIRO trial randomised 207 patients to undergo traditional open TTO or a hybrid MIO approach comprising a laparoscopic abdominal phase combined with an open thoracotomy [15]. The primary outcome of major 30 day post-operative complications was significantly decreased, from 64 to 36% (*P* < 0.001), using the hybrid approach, mainly relating to decreased major respiratory complications.

**Table 2:** Key randomised controlled trial (RCT) evidence base comparing minimally invasive oesophagectomy (MIO), a hybrid approach, and robot-assisted minimally invasive oesophagectomy (RAMIE) with open transthoracic surgery.

Trial	Comparison	Arm A	Arm B	*P*	Other comments
MIRO [15] *N* = 207	Arm A: open TTO Arm B: Hybrid 2 resection	64% major complications 30% pulmonary complications	36% major complications 18% pulmonary complications	<0.001	Trends towards improved survival with hybrid approach, 3-year survival 67% hybrid versus 55% open, NS
TIME [102] *N* = 115	Arm A: MIO Arm B: open TTO	34% pulmonary infection (at 30 d)	9% pulmonary infection (at 30 d)	0.005	3-year survival 51.5% MIO versus 40.4% open (*P* = 0.02)
ROBOT [16, 108] *N* = 112	Arm A: open TTO Arm B: RAMIE	80% overall complications	59% overall complications	*P* = 0.02	Reduced blood loss (*P* < 0.001) and pulmonary complications (*P* = 0.005), and improved functional recovery (*P* = 0.03) with RAMIE. 5-year survival 41% RAMIE, 40% open, NS

*NS*   non-significant, *TTO* transthoracic oesophagectomy.

However, some issues remain. In the TIME trial, the primary endpoint of proven pneumonia in the open group was higher than anticipated and previously reported [101, 102]. In MIRO, Clavien-Dindo grade II severity complications were defined as ‘major’, which might not be commonly accepted, as Grade II do not require surgical or radiological intervention; and the results were equivalent for both treatment arms with respect to ≥ grade III severity complications, suggesting that hybrid oesophagectomy may have a more significant impact on less severe complications [15]. A further caveat comes from a registry study from the Netherlands comparing MIO and open approaches, in which an increase in perioperative complications, reoperation rates and length of stay for patients undergoing MIO was reported [101]. This discordance might reflect a learning curve for those training in MIO, or could relate to low-volume centres performing procedures, but it is clear that further real-world data and rigorous clinical audit are necessary to ensure patient outcomes are not compromised by the introduction of new surgical techniques [103]. With respect to current RCTs, no direct comparison of a hybrid approach and a totally MIO approach is currently being investigated. The ROMIO trial aims to compare open, hybrid and MIO approaches in over 400 patients, with a primary outcome of patient-reported physical status post operatively [104].

## Robot-assisted approaches

Throughout most of surgery, robotic-assisted approaches are undergoing testing and evaluation, while modern trainees are increasingly focused on acquiring robotic skills and accreditation. Advantages of a robotic approach might include articulation of the instruments, minimisation of large movements for the surgeon, and better ergonomics and, arguably, this approach might supplant minimally invasive approaches if the considerable costs can be contained. In oesophageal cancer surgery, a robotic approach was first described by Kernstine et al. [105], and advanced in Europe mainly by Professor Van Hillegersberg and colleagues in Utrecht, the Netherlands, and this field of robotic assisted minimally invasive oesophagectomy (RAMIE) has progressed rapidly in the past decade [16, 105]. The ROBOT Trial compared robot-assisted three-stage oesophagectomy and open three-stage oesophagectomy in a study of 109 patients, with a primary endpoint of post-operative complications [16]. RAMIE was associated with decreased overall complications (59% versus 80%, *P* = 0.02), as well as decreased blood loss, reduced pain and earlier functional recovery, with 5-year survival equivalent between the two approaches, at 42% versus 43% (*P* = 0.74), for RAMIE and open, respectively [16, 106].

A caveat of a robotic approach as for MIO, relates to anastomotic leak rates, and highlights the need for rigorous mentoring and accreditation, and oversight in training, in the future. A 2020 international registry study reported leak rates of 33% for robot-assisted hand-sewn anastomosis, 17% for circular stapled anastomosis and 15% for linear stapled anastomosis using RAMIE during a particular procedure [107]. These rates are high, and well above reported rates from high-volume centres with standardised stapled or hand-sewn techniques [101]. To this end, the OesophagoGastric Anastomosis Audit examining oesophago–gastric anastomosis might provide some clarity as to the optimal anastomotic techniques in standard oesophagectomy, MIO and RAMIE [108].

## Conclusion

Despite the considerable challenge in curing a cancer that frequently presents at an advanced stage and that might have adverse biological features that promote resistance to standard therapies, significant progress has been made in the curative approach to oesophageal cancer. An increasing reserve of RCTs nearing completion, as well as advances in endotherapy, staging, surgery and high-content scientific analysis, in addition to an improved understanding of genomics and the tumour microenvironment offers a real promise of further discovery and improved cure rates. The model for the curative approach to locally advanced oesophageal cancer is likely to change in the not-too-distant future. Information on HER2 status, PD-L1 expression, MSI and overall mutational burden—currently the preserve of metastatic disease algorithms—might be increasingly relevant for the incorporation of targeted therapy within treatment plans, and to help guide the response to therapy [94, 109, 110]. Challenges exist, with few targetable mutations in oesophageal cancer, and a high mutational frequency in highly heterogenous tumours. Cost is also an issue, as is affordable, efficient high-throughput sequencing with a clear clinical application whether in stratifying prognosis or in tailoring therapies. Biomarkers are also needed and, in this context, circulating tumour DNA has shown some promise [111, 112]. As technology and techniques evolve in EET and surgery, including the potential for increased use of artificial intelligence, there may be significant challenges in the introduction of this technology. In addition to significant cost implications, there must be equitable access to novel techniques to ensure patients benefit in a just way. This review, written mainly from the perspective of academic surgeons and oncologists, although focused on five major themes, highlights key advances in medicine and science, and novel vistas that continue to give cause for optimism.

## Data Availability

Not applicable.
